# Parasite distribution and associated immune response during the acute phase of *Toxoplasma gondii* infection in sheep

**DOI:** 10.1186/s12917-014-0293-5

**Published:** 2014-12-16

**Authors:** Delfien Verhelst, Stéphane De Craeye, Gary Entrican, Pierre Dorny, Eric Cox

**Affiliations:** Laboratory of Immunology, Faculty of Veterinary Medicine, Ghent University, Ghent, Belgium; National Reference Laboratory for Toxoplasmosis, Operational Direction Communicable and Infectious Diseases, Scientific Institute of Public Health, Brussels, Belgium; Moredun Research Institute, Pentlands Science Park, Bush Loan, Midlothian, Penicuik, EH26 0PZ Scotland UK; Department of Biomedical Sciences, Institute of Tropical Medicine, Antwerp, Belgium; Laboratory of Parasitology, Faculty of Veterinary Medicine, Ghent University, Ghent, Belgium

**Keywords:** *Toxoplasma gondii*, Immune responses, Quantitative PCR, ELISA, Sheep

## Abstract

**Background:**

In many countries, *Toxoplasma gondii* (*T. gondii*) is a major cause of reproductive disorders and abortions in the sheep industry, and therefore responsible for important financial and economic losses. In addition, undercooked infected lamb is an important risk factor for human toxoplasmosis.

In the present study, the initial phase of the infection was investigated: the parasite’s entry site, the subsequent distribution of the parasite and the host-immune response.

**Results:**

Parasite DNA was already detected in the cranial small intestinal mucosa the first days after oral infection with *T. gondii* tissue cysts. Simultaneously, high IFN-gamma and IL-12 responses were induced mainly in the mesenteric lymph nodes. The emergence of IgG1 (at 8dpi), and IgG2 (at 11 dpi) was accompanied by a decrease or even disappearance of the IFN-gamma and IL-12 response in the Peyers’ patches (PP), PBMC’s and popliteal LN’s. Meanwhile the parasite DNA could be recovered from most mucosal and systemic tissues to become undetectable in the small intestine, popliteal LN, PBMC and spleen 3 weeks pi.

**Conclusions:**

Our results indicate that parasites enter the cranial small intestine the first days after infection and that after an increase the first two weeks after infection, the parasite DNA levels in the intestine drop below the detection limit three weeks after infection. This coincides with an increase in parastic-specific serum IgG1 and IgG2 and a decrease of the antigen-specific IFN-gamma response in PP, PBMC and popliteal LN. We suggest a role for IFN-gamma and IL-12 in controlling the infection.

## Background

*Toxoplasma gondii* is an obligate intracellular parasite with a worldwide prevalence in a wide variety of hosts [1]. In many countries, the parasite is a major cause of reproductive disorders, miscarriages and abortions in the sheep industry, and therefore responsible for important financial and economic losses [2,3]. Based on data collected by the Veterinary Investigation Diagnosis Analysis in 2009 [4], toxoplasmosis is the second cause of abortions in sheep in the UK after *Chlamydia abortus* (45.6%).

Undercooked infected lamb is considered as an important risk factor for human toxoplasmosis [5]. Infection of herbivorous animals occurs mostly through ingestion of *T. gondii* oocysts excreted by cats. One week after a first infection, cats can shed over 100 million oocysts in their faeces during a period of 7–14 days depending on the infection dose, the infection stage and the immune status of the cat. Sporulated oocysts can stay infectious in the environment during more than 1 year and contaminate pastures, feeds and also drinking water [6,7].

An acute primary infection during gestation will result in transmission of the parasite to the foetus. As in humans, the consequences for the foetus depend on the stage of the gestation: an early infection (during the first or second trimester of gestation) results in fetal death and resorption or abortion, whereas a later infection leads to the birth of clinically normal but latently infected lambs or of lambs with symptoms of congenital toxoplasmosis (fever, growth retardation, weakness) [8-10].

The immunity developed by the pregnant ewe will protect it against infection of the foeti during subsequent gestations [7]. The same protection seems to occur when a non-pregnant ewe becomes infected with *T. gondii* [10,11]. Data of Morley et al. [12,13] suggested that immunity might protect less well against transplacental transmission than thought. However, their studies were based on *T. gondii* DNA detection only and not on demonstration of *T. gondii*-associated lesions by histopathology, nor did they exclude other abortifacient agents [14]. Williams et al. [15] suggest that the observations of Morley et al. [12,13] are due to breed differences. Furthermore, the effectiveness of vaccination with Toxo-Vax is mentioned in several studies [16-18].

Whereas several experimental infections have been carried out in sheep to study host-immune responses, to our knowledge, the distribution of the parasite and the correlated antibody and interferon (IFN)-gamma responses during acute ovine toxoplasmosis have not been studied yet [2,7,14,19-21].

In the present study, oral infections of sheep with *T. gondii* tissue cysts were performed to determine the entry site and subsequent distribution of the parasite following the natural infection route. Furthermore, we monitored the local IFN-gamma response during the acute phase of the infection, because of its important role as a mediator of host resistance against *Toxoplasma* as demonstrated in mice, sheep and humans [17,22,23].

## Methods

### Animals and experimental procedure

Thirteen 7-week-old conventionally reared lambs were selected (Belgian cross-breed, Zootechnical Centre, Leuven, Belgium), based on their *T. gondii* and *Neospora caninum* seronegative status. The absence of antibodies against *T. gondii* was assessed by an indirect immunofluorescence assay (IFA, Toxo-Spot IF, Biomérieux, Marcy-l’Etoile, France). As anti-*Neospora* antibodies could cross-react with *T. gondii* antigens, the sera were also tested with the *Neospora caninum* Antibody Test Kit (CHEKIT* Neospora, Idexx, Hoofdorp, The Netherlands).

After an acclimation period of one week at the experimental animal facilities of Ghent University, eleven animals were orally infected with 3000 tissue cysts of the *T. gondii* Prugniaud strain (PRU). PRU is a type II genotype isolated from a case of human lethal congenital toxoplasmosis [24,25]. Sheep were killed by captive bolt and exsanguination on day 4 (4 dpi; N = 3), 8 (8 dpi; N = 2), 10 (10 dpi; N = 2), 14 (14 dpi; N = 1) and 21 (21 dpi; N = 3) after infection. Two animals were kept as negative controls (C) and euthanized at 21 days. Blood samples were taken daily from the jugular vein of each animal until euthanasia for monitoring the *T. gondii*-specific serum antibody response. At euthanasia, portions of duodenal (D), jejunal (J) and ileal (I) tissue with and without Peyer’s patches (PP), the draining mesenteric lymph nodes (MLN), popliteal lymph nodes, spleen and blood on heparin were sampled. Samples were processed as described further to determine the spread of the parasite as well as the appearance of a specific IFN-gamma response in the different tissues at the early phase of the infection.

All experimental and animal management procedures were approved by the Animal Care and Ethics Committee of the faculties of Bioscience Engineering and Veterinary Medicine, Ghent University (2007/103).

### Inoculum

Tissue cysts of the *T. gondii* PRU strain were harvested from the brains of chronically infected C57BL6 mice and quantified as described by Verhelst *et al.* [26]. Briefly, infected mice were euthanized five weeks after infection and their brains were homogenized in phosphate buffered saline (PBS) using a Potter Homogenizer. The concentration of brain cysts in the suspension was determined by phase contrast microscopy whereafter the brain homogenate was diluted in PBS to obtain a final concentration of 300 cysts per ml. The sham inoculate given to the control animals was prepared similarly from brains of non-infected mice.

### Purification of recombinant antigens

The recombinant antigens GRA7, EC2 and MIC3_234–307_ were used to stimulate cell suspensions for IFN-gamma and IL-12 production. GRA7 has already been demonstrated to be useful as a sero-diagnostic marker of acute and chronic toxoplasmosis [27-29]. Furthermore GRA7 is expressed by all infectious stages of *T. gondii* and appears plentiful on the surface of the host cells, the host cell cytosol, the parasitophorous vacuolar membrane and lumen [30]. After rupture of the host cells, high amounts of GRA7 come in contact with the host immune system, triggering strong antibody responses [31]. The recombinant EC2 antigen is a chimer of the antigenic regions of the microneme proteins MIC2 and MIC3 and of the surface antigen SAG1.

The recombinant antigens were produced as previously described [26,32]. Briefly, the HIS-tagged recombinant antigens were purified from an *E. coli* culture using metal chelate affinity chromatography (Ni-NTA Superflow, Qiagen, GmbH, Hilden, Germany), according to the manufacturer’s instructions. The recombinant antigens were eluted from the Ni-NTA columns by incubation in 500 mM imidazol buffer followed by dialysis through a CelluSep H1 membrane with a 8 kDa cut-off (Membrane Filtration Products, Texas, USA) in urea buffer for EC2 and GRA7 and in PBS for MIC3_234–307_.

*Toxoplasma* total lysate antigen (TLA) was produced as described before [33]. Briefly, *T. gondii* RH tachyzoites (type I strain) were harvested from the peritoneal cavity of Swiss mice, which had been intraperitoneally infected 4 days earlier. Hereto, collected ascites fluid was passed twice through a 26-gauge needle. After centrifugation for 15 minutes at 950 × g, the pellet containing the tachyzoites was washed twice in PBS, followed by sonication in an Ultrasonic disintegrator (MSE, Leicester, United Kingdom) to solubilize the *T. gondii* tachyzoite antigens. The total protein concentration was measured by the bicinchoninic acid (BCA) reaction (Thermo Scientific Pierce BCA Protein Assay Kit), whereafter the antigen suspension was aliquoted and stored at −20°C until use.

### Indirect Immunofluorescence Assay (IFA)

To test for the presence of specific IgG and IgM antibodies against *T. gondii;* the sheep sera were diluted 1:50 in PBS and tested as follows: fifty microliter of this dilution was applied on a slide coated with formalin-treated *T. gondii* RH tachyzoites (Toxo-Spot IF, Biomérieux, Marcy-l’Etoile, France) for 30 min at 37°C. On each slide, seronegative and seropositive sheep reference serum samples were used as controls. After washing with PBS, 30 μl of a 1:25 in PBS-Evans Blue diluted fluorescein isothiocyanate conjugated rabbit anti-sheep IgM or rabbit anti-sheep IgG (KPL, Maryland, USA) was applied for 30 minutes at 37°C. The slides were then washed again, air-dried and read using a fluorescence microscope.

### T. gondii -specific antibody ELISA

The presence of *T. gondii* antigen-specific IgG, IgG1, IgG2 and IgA in the collected sera was tested by ELISA. Nunc maxisorp immunoplates (Life Technologies, Merelbeke, Belgium) were coated overnight at 4°C with TLA at a concentration of 10 μg/ml in bicarbonate coating buffer (pH 9.7). In subsequent steps, the plates were blocked during 2 hours at 37°C in 1% non-fat milk powder in PBS, incubated for 1 h at 37°C with 100-fold diluted sera in PBS with 0.05% Tween® 20 and 1% non-fat milk powder. This was followed by an incubation step for 1 h at 37°C with rabbit anti-sheep IgG- or IgA-labelled with horseradish peroxidase (AbD Serotec (Gentaur), Brussels, Belgium); or with a mouse anti-bovine IgG1 or IgG2 secondary antibody followed by an incubation with an anti-mouse immunoglobulin-horseradish peroxidase conjugate. Subsequently a 3,3′,5,5′-tetramethylbenzidine solution (TMB)(Sigma–Aldrich, Diegem, Belgium) was added. In between each step, plates were manually washed 5 times with PBS 0.05% Tween® 20. The reaction was stopped by adding 50 μl 2 M H_2_SO_4_ (stop solution). Absorbance was read at 450 nm. Positive and negative control sera were included on each plate. The corrected optical density (OD) was calculated as: OD of sample – OD of negative control sample. The serum samples were considered positive when the corrected optical density (OD_450_) of the dilutions exceeded the cut-off value (= mean OD_450_ (negative controls) + 3 × its standard deviation). The negative control sera in the IFA and ELISA were from *T. gondii* negative sheep. They tested negative in the Modified Agglutination test, the IFA and the Sabin Feldman Dye Test.

### Isolation of mononuclear cells (MCs) from blood, spleen, lymph nodes and intestine

The MCs from blood and spleen were isolated as described by Verhelst *et al*. [26]. The peripheral blood MCs (PBMCs) were isolated by density gradient centrifugation using Lymphoprep™ (Nycomed, Brussels, Belgium). Briefly, heparinized blood was centrifuged during 25 min at 1000 × g and 18°C. After removing the plasma layer, the buffy coat and erythrocyte pellet were suspended in an equal volume of PBS with 1 mM ethylediaminetetraacetic acid (PBS-EDTA) and layered onto the gradient. Tubes were centrifuged for 45 min at 800 × g and 18°C. The interface layer, containing the MCs, was collected and the cells were washed in PBS-EDTA.

Splenocytes were isolated from the spleen after removing the surrounding fat. The obtained cell suspension was further purified by lysing the erythrocytes in the presence of ammonium chloride. After centrifugation (380 × g at 18°C for 10 min), the pelleted cells were washed and resuspended in PBS-EDTA (1 mM).

The MCs from mesenteric lymph nodes (MLN), popliteal LN, duodenal, jejunal and ilial Peyers Patches (PP) were isolated as described by Vandenbroeck *et al*. [34] and the MCs from the duodenal, jejunal and ileal lamina propria as described by Vande Walle *et al*. [35]. All isolated cells were resuspended at a final concentration of 1 × 10^7^ cells/ml in complete leucocyte medium (RPMI 1640, Gibco, Merelbeke, Belgium) supplemented with 10% fetal calf serum (FCS, Greiner Bio-One, Belgium), 200 mM l-glutamin (Gibco), 100 U/ml penicillin and 100 μg/ml streptomycin (P/S; Gibco), 100 mM non-essential amino acids (Gibco) and 100 μg/ml kanamycin (Gibco).

### Isolation of enterocytes from the intestine

At euthanasia, duodenal, jejunal and ileal segments without PP of approximately one meter were sampled, flushed three times with Krebs buffer (0.12 M NaCl, 0.014 M KCl, 0.001 M KH_2_PO_4_, 0.025 M NaHCO_3_, pH 7.4) at room temperature and filled for 95% with Hank’s Buffered Salt Solution (HBSS) supplemented with 1.5 mM EDTA +1 mM 1,4-dithiothreitol (DTT) pre-heated at 37°C. Then the segments were incubated in 2 liter PBS for 15 min at 37°C. Thereafter, the contents were collected and placed at 4°C. This process was repeated twice. The isolated cells were pooled and washed three times with HBSS supplemented with 0.1 mM phenylmethylsulfonyl fluoride (PMSF). PMSF is a serine protease inhibitor that prevents proteolytic degradation of proteins [36]. After centrifugation for 10 min at 1800 × g and 4°C, the cells were resuspended in complete medium at a concentration of 10^7^ cells/ml.

### Detection of parasite DNA in cell populations by real-time PCR

DNA was extracted from cell suspensions using the Qiagen QIAmp DNA Mini kit (Qiagen GmbH, Hilden, Germany), according to the manufacturer’s instructions. Next, the presence of *T. gondii* DNA was determined by duplex qPCR analysis as described before [37]: the *T. gondii* repeat element (AF146527) was used as target for the detection of the parasite’s DNA and the amplification of the cellular 18S rRNA gene Since host cells are always present in the sample, the latter reaction should always be positive. Tenfold dilutions of a counted number of RH tachyzoites and cultured swine kidney cells (SK-6) were used to generate a standard line for the quantification of *T. gondii* parasite DNA and one for the cells, respectively. Real-time PCR was performed with an initial 3 min denaturation step at 95°C, followed by 45 cycles at 95°C for 15 sec and 60°C for 20 sec.

### Restimulation of isolated mononuclear cells with T. gondii antigens

The MCs were seeded in 96-well flat bottom cell culture plates (Greiner bio-one) at 10^6^ cells/ per 100 μl complete medium and per well. After an initial incubation of 12 h, the mitogen concanavalin A (Con A) (5 μg/ml) (BD Biosciences) or 10 μg/ml of one of the following antigens was added: rEC2, rGRA7, rMIC3 or, total lysate antigen (TLA). The recombinant proteins were diluted in complete medium, supplemented with 10 μg/ml polymyxin B to neutralize potentially contaminating endotoxin [38]. After a 6 h incubation at 37°C in a humidified atmosphere with 5% CO_2_ for Con A or 96 h for the antigens, the cell-free supernatant was removed and stored at −20°C until analysed for interferon-gamma (IFN-gamma) concentration by ELISA.

### Cytokine ELISA

To determine the IFN-gamma and interleukin 12 (IL-12) concentrations in the cell-free culture supernatants, 96-well Nunc maxisorp immunoplates (Life Technologies) were coated overnight at 4°C with a mouse anti-bovine IFN-gamma monoclonal antibody (mAb) (CC330; AbD Serotec) at 5 μg/ml or with a mouse anti-bovine IL-12 mAb at 2.5 μg/ml (CC301; AbD Serotec), respectively. Then, the plates were blocked for 2 h at room temperature (RT) with PBS containing 0.05% Tween® 20 and 3% non-fat milk powder. In subsequent steps we added each for 1 h at RT: 100 μl of cell culture supernatant 1:2 in PBS supplemented with 0.05% Tween® 20 and 1% non-fat milk powder, a biotin-labelled mouse anti-bovine IFN-gamma mAb (CC302b; AbD Serotec) or a biotin-labelled mouse anti-bovine IL-12 mAb (CC326; AbD Serotec) and horseradish peroxidase (HRP)-conjugated streptavidin (Abd Serotec). In between each step, plates were washed 5 times with PBS supplemented with 0.05% Tween® 20. Then, a HRP substrate solution (3,3′,5,5′-tetramethylbenzidine (TMB, Sigma – Aldrich)) was added for 30 min at RT in the dark. The reaction was stopped by adding 50 μl H_2_SO_4_ (2 N) and the optical density (OD) was measured at 450 nm.

The IFN-gamma and IL-12 concentrations in the cell culture supernatants were calculated from a regression line using the DeltaSoft JV 2.1.2 software. The regression lines were obtained by including in the tests 10-fold dilutions of recombinant ovine IFN-gamma or IL-12 starting from 4000 pg/ml and 320 u/ml, respectively. The recombinant ovine IFN-gamma and ovine IL-12 were prepared as a serum-free conditioned medium from transfected CHO cells, according to a protocol described for the production of recombinant ovine IL-4 [39]. The recombinant ovine IFN-gamma was quantified using the recombinant bovine IFN-gamma standard sold by Endogen, Thermo-scientific (Rockford, USA) and the anti-bovine mAbs clones CC330 and CC302b (AbD Serotec) in a species cross-reactive ELISA (personal communication with Sean Wattegedera, Moredun Research Institute).

## Results

### Neospora caninum antibodies

None of the control or experimentally infected animals showed IgG response against *N. caninum* at euthanasia*.*

### Presence of T. gondii in tissues

The presence of *T. gondii* in the various tissues collected after euthanasia was evaluated by qPCR (Table 1). At 4 days post infection (dpi), parasite DNA could be found sporadically in tissues of two out of three sheep. These sheep had *T. gondii* DNA in their epithelium (enterocytes, lamina propria) and in the organized lymphoid tissue of the small intestine (PP) or draining the small intestine (MLN), mostly in the cranial parts. In one sheep the parasite could be detected in peripheral blood mononuclear cells (PBMC). In the third sheep, no *T. gondii* DNA was detected, suggesting that the parasite had not yet invaded the gut at this time point, had already left this site, or that the parasitic load was still below the detection limit. In the sheep analysed at 8 dpi, more tissues were found positive for *T. gondii* DNA. The epithelium and draining lymph nodes of both sheep were positive throughout the small intestine, the lamina propria and Peyer’s patches in the duodenum. Other parts of the small intestine, popliteal lymph nodes and spleen were positive in only one sheep. This presence in the systemic tissues indicates dissemination was occurring. At 10 dpi a similar pattern was seen for the intestinal tissues and MLN, but now both euthanized animals showed parasite DNA in their popliteal lymph nodes, confirming the dissemination observed in one sheep at day 8. At 14 dpi the only euthanized animal had parasite DNA in all its intestinal tissues with the highest amounts in the Peyer’s patches and MLN, whereas popliteal lymph nodes and spleen were negative. One week later (21 dpi), the parasite load had dropped below the detection limit in most tissues. In one animal only parasite DNA was demonstrated in its MLN with the highest amounts in jejunal MLN. The other animal had low levels of parasite DNA in its jejunal epithelium and duodenal lamina propria. The third sheep tested negative. This suggests that either most of the parasites had left the intestinal and lymphoid tissues or were cleared from these tissues at this time point.

**Table 1:** **Presence of parasite DNA in different small intestinal tissues, lymph nodes and blood of sheep orally infected with 3000** ***T. gondii*** **PRU tachyzoites and euthanized at different days post infection (dpi)**

**Time**	**Parasite load as determined by RT qPCR and expressed as log 10 of parasite DNA/10** ^**6**^ **cells**											
	**DPI**	**4**			**8**		**10**		**14**	**21**		
	**Animals**	**Sh1**	**Sh2**	**Sh3**	**Sh4**	**Sh5**	**Sh6**	**Sh7**	**Sh8**	**Sh9**	**Sh10**	**Sh11**
Duodenal E		0,36	nd	nd	3,06	-2,26	-3,06	-2,23	-2,90	nd	nd	nd
Duodenal LP		-0,43	nd	nd	2,88	-1,60	-1,46	-2,59	2,74	nd	nd	-1,58
Duodenal PP		nd	0,11	nd	2,26	-1,15	-2,68	-0,74	4,09	nd	nd	nd
Jejunal E		nd	nd	nd	2,23	-0,64	nd	-2,67	-1,54	nd	nd	-0,67
Jejunal LP		nd	nd	nd	nd	-0,02	-1,67	nd	-1,96	nd	nd	nd
Jejunal PP		nd	nd	nd	nd	-1,23	nd	-2,07	3,40	nd	nd	nd
Ileal E		nd	-0,16	nd	0,87	-1,44	nd	-3,29	-1,86	nd	nd	nd
Ileal LP		nd	nd	nd	nd	-0,44	-1,91	-4,00	-2,10	nd	nd	nd
Ileal PP		nd	nd	nd	nd	-2,00	nd	nd	2,95	nd	nd	nd
Duodenal MLN		0,34	nd	nd	1,95	-0,31	-1,20	-0,77	2,76	0,05	nd	nd
Jejunal MLN		2,63	nd	nd	1,45	-0,51	-0,99	-0,94	3,67	8,37	nd	nd
Ileal MLN		nd	nd	nd	2,44	-0,72	-2,87	-1,06	3,19	-1,29	nd	nd
Peripheral blood MC		0,45	nd	nd	nd	nd	nd	-1,68	-1,30	nd	nd	nd
Popliteal LN		nd	nd	nd	nd	-1,54	-1,66	-0,89	nd	nd	nd	nd
Splenocytes		nd	nd	nd	nd	-2,49	nd	-2,00	nd	nd	nd	nd

Sh = Sheep. E = Enterocytes. LP = Lamina propria. PP = Peyers’ patches. MLN = Mesenteric lymph nodes. MC = Mononuclear cells. LN = Lymph node. Nd = Not detected.

When comparing the parasitic load in the tissues of the four animals euthanized at 8 and 10 dpi a trend could be observed in cranial versus caudal intestinal tissues. For each of the four animals, one or more of the tested duodenal tissues or its associated draining lymph node, showed the highest or second highest amount of parasite DNA, while the tested ileal tissues often showed the lowest amount. In 5 of 12 ileal tissues samples even no parasite DNA could be detected at all. Overall the ileal Peyer’s patches showed the lowest amount of parasite DNA. These data suggest that shortly upon release from the cysts, the parasite invades the gut primarily in the cranial small intestine.

The two sham infected animals remained *T. gondii*-free in all tissues.

### Humoral immune response

**Figure 1:**
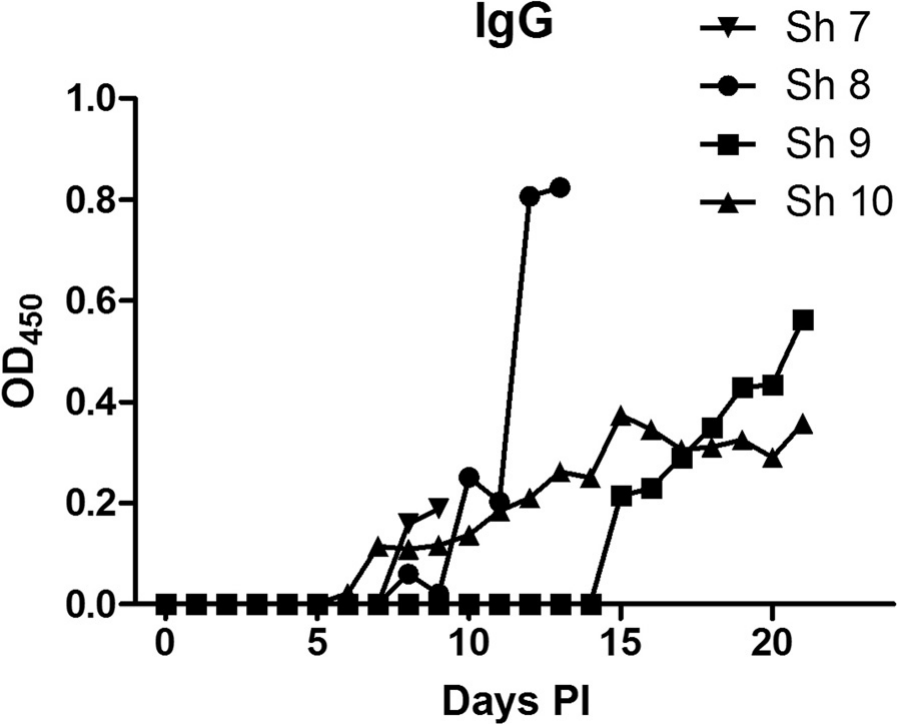
**IgG response of the sheep in TLA ELISA in function of time.**

The kinetics of the antibody response after the oral infection was determined on sera collected daily. Both a *Toxoplasma*-specific IgM and IgG IFA as a TLA-specific IgG, IgG1, IgG2 and IgA ELISA were performed. Data of the TLA-specific IgG ELISA are presented in Figure 1.

*Toxoplasma*-specific IgM was first detected at 9, 12 and 15 dpi, in three of the then 6 non-euthanized sheep (3/6), two on 4 sheep (2/4) and two on 3 sheep (2/3), respectively. In one sheep no specific IgM could be detected until euthanasia at 3 weeks post infection. IgG was detected by IFA in one sheep at 9 dpi (1/6) and in another sheep at 12 dpi (1/4). These animals remained seropositive until euthanasia.

In ELISA, the *T. gondii*-specific IgG was detected first at 7, 8, 10 and 15 dpi, each time in one animal and remained present in these animals until euthanasia. Interestingly, in one animal no TLA-specific IgG response was seen although parasite DNA could be detected in its tissues at euthanasia 21dpi. IgG1 appeared in one sheep 15 dpi and in another sheep 17 dpi. IgG2 appeared in the same two animals, but slightly earlier, namely 12 and 15 dpi. The two sham infected control sheep did not show *T. gondii-specific* antibodies in IFAT, nor in ELISA.

### Interferon-gamma and interleukin-12 cytokine response

At euthanasia, MCs were isolated from blood, Peyer’s patches, MLN, popliteal lymph nodes and spleen and restimulated with rGRA7, rEC2, rMIC3 and total lysate antigen (TLA) to determine the antigen-specific IFN-gamma and/or IL-12 responses.

**Figure 2:**
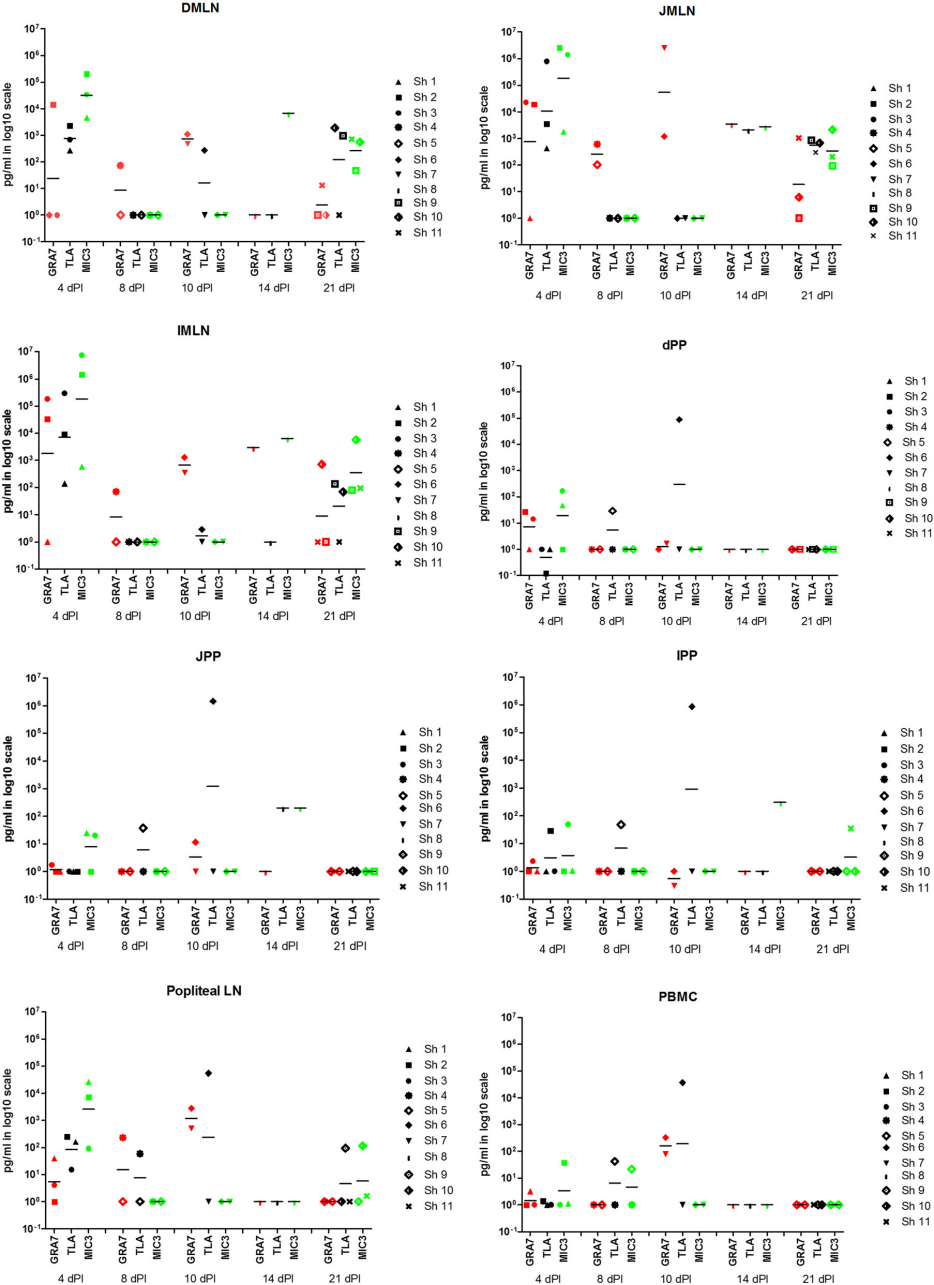
**IFN-gamma response (log10 scale) in mesenteric lymph nodes, Peyer’s Patches, popliteal LN and PBMC against GRA7, TLA and MIC3 in function of time.**

At 4 dpi, TLA, EC2 and MIC3 consistently induced high IFN-gamma concentrations in the MLN cultures of all 3 animals, with the highest responses against MIC3 (DMLN: 80,637 ng/ml (mean) ± 61,951 (SEM); JMLN: 1,313,771 ng/ml ± 735,659; IMLN: 2,963,760 ng/ml ± 2,275,224), followed by EC2 (DMLN: 5,400 ng/ml ± 2,204; JMLN: 129,964 ng/ml ± 119,802; IMLN: 298,194 ng/ml ± 155.314) and TLA (Figure 2). In contrast, GRA7 less consistently induced IFN-gamma (Figure 2): 2 out of 3 animals showed IFN-gamma production in jejunal and ileal MLN and 1 out of 3 in duodenal MLN. IFN-gamma was also induced in popliteal LN and spleen, but mainly by MIC3 and to a lesser extent by TLA. Responses in PP and PBMC were absent.

At 8 and 10 dpi, the IFN-gamma pattern was completely different with mostly low or no responses in MLN, but responses more localized in PP, PBMC and popliteal LN with the highest concentrations following TLA stimulation and lower responses following GRA7 stimulation. Neither EC2, MIC3 nor TLA induced IFN-gamma responses in the MLN cell cultures. Only GRA7 induced IFN-gamma in MLN of all 4 animals with the lower concentrations at 8 dpi (Figure 2). GRA7 also induced IFN-gamma production in popliteal LN cell cultures of 3 out of 4 animals, and in PBMC and spleen cell cultures in 2 out of 4 animals, but it did not induce IFN-gamma in PP cell cultures. Only TLA induced IFN-gamma in PP cell cultures of all 3 small intestinal sites in 2 out of 4 animals. The same 2 animals showed TLA-induced IFN-gamma in popliteal LN and PBMC cultures. At 14 and 21 dpi, the IFN-gamma response was also pronounced in the MLN and to a lesser extent in the spleen, with mainly MIC3 inducing good responses in MLN, whereas especially TLA gave high responses in the spleen. TLA and GRA7 induced-responses were absent in PP, popliteal LN and PBMC cultures, whereas low TLA induced-responses could be seen in spleen cell cultures of 3 on 4 animals (one sheep euthanatized at 14 dpi and 2 at 21 dpi) and low MIC3 and EC2-induced-responses in 2 on 4 animals (at 21 dpi). All 4 animals showed IFN-gamma responses induced by TLA, EC2, MIC3 and GRA7 in at least one of their MLN cultures, but responses were lower than those seen at 4 dpi. There was no IFN-gamma induced in the MCs of the control animal.

**Figure 3:**
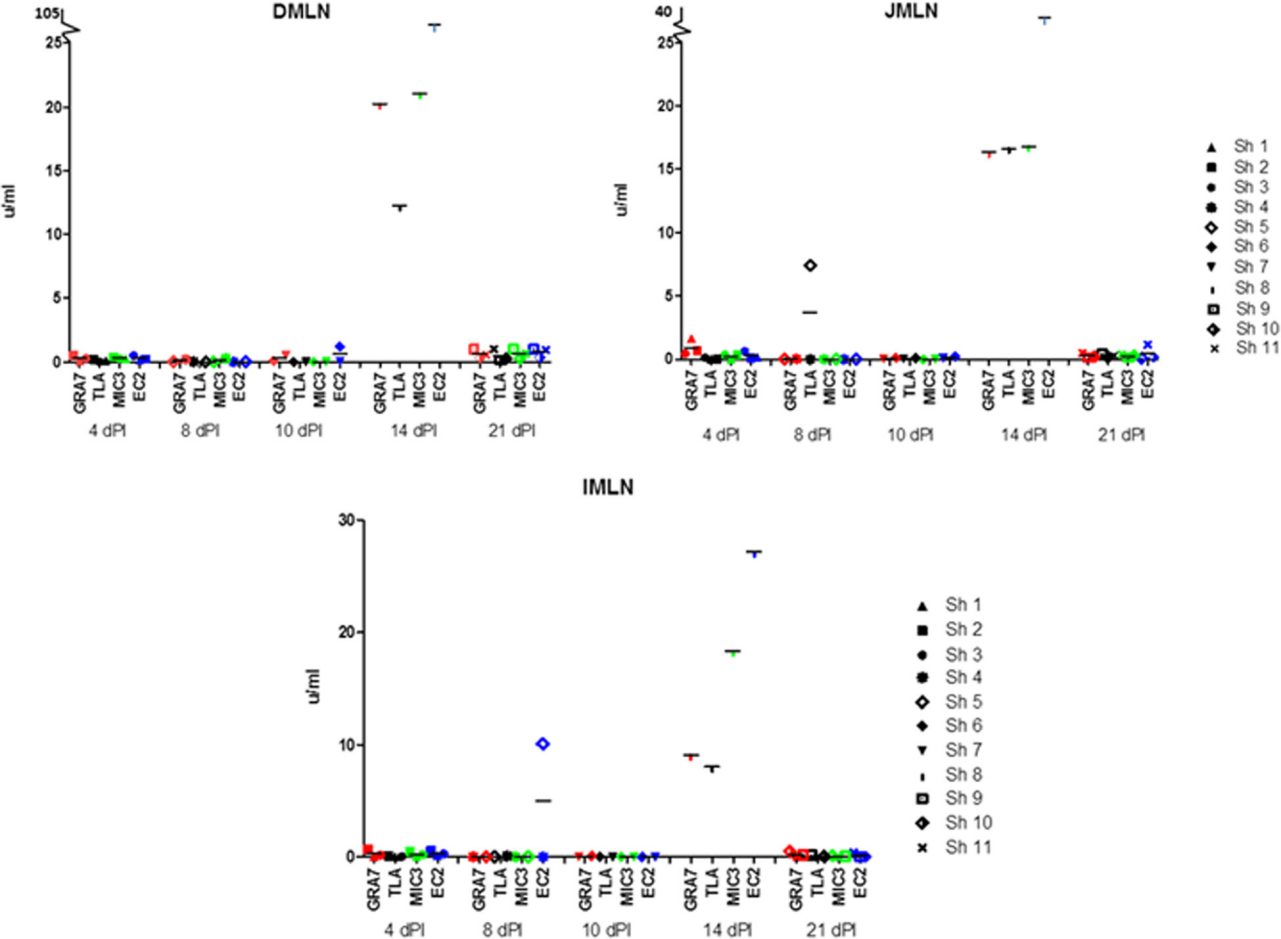
**IL-12 response (u/ml) in mesenteric lymph nodes against GRA7, TLA, MIC3 and EC2 in function of time.**

Comparable to IFN-gamma, IL-12 production at day 4 was most consistently induced in the MLN cell cultures (Figure 3). However, whereas GRA7 was the weakest inducer of IFN-gamma, in JMLN it was the best inducer of IL-12 (0.50 u/ml ± 0.16 (SEM)), followed by EC2 (0.29 u/ml ± 0.07 (SEM)), MIC3 (0.26 u/ml ± 0.06 (SEM)) and TLA (0.06 u/ml ± 0.02 (SEM)). The latter induced low amounts of IL-12 in DMLN of one animal and in JMLN of another animal. Besides, low amounts of IL-12 were induced by MIC3 in PP of two animals, by TLA in the PP of the third animal and by EC2 in the popliteal LN of one animal.

At 8 and 10 dpi, the responses of MLN were overall lower except for one animal in which TLA induced 7.4 u/ml in jejunal MLN and EC2 10.1 u/ml in ileal MLN. Neither of the antigens consistently induced IL-12 responses in the MLN cell cultures, but in each animal at least one antigen induced IL-12 in at least one of the MLN cell cultures (Figure 3). The same was seen for the PP cell cultures. In popliteal LN cell cultures and spleen, two of the 4 animals showed IL-12 production following restimulation with different antigens. PBMCs remained IL-12 negative. So at these time points more variation was seen in the IL-12 response than in the IFN-gamma response.

At 14 dpi, EC2 induced IL-12 in cell cultures of all tissues except of spleen, with high concentrations in duodenal MLN (40.9 u/ml) and extremely high concentrations in popliteal LN cell cultures (635,62 u/ml). None of the other antigens induced IL-12 in MLN cell cultures (Figure 3). For the PP, GRA7 and MIC3 induced IL-12 in duodenal, EC2 in jejunal and MIC3 and TLA in ileal PP cell cultures. Besides EC2, TLA induced also high IL-12 concentrations in the popliteal LN cell cultures (32.8 u/ml). MIC3, GRA7 and TLA induced IL-12 in PBMCs and splenocytes (1.74 – 6.08 u/ml). So, especially the IL-12 response in popliteal LN and PBMCs was a noteworthy difference with the IFN-gamma response.

At 21 dpi, the IL-12 response was absent in popliteal LN and PBMCs, was present in the splenocytes culture of one animal stimulated with EC2 and MIC3 and was consistently present in low concentration in duodenal and jejunal MLN cultures stimulated with the recombinant antigens and TLA (Figure 3). It was less consistently present in ileal MLN cultures, similar to the IFN-gamma response.

## Discussion

*T. gondii* seropositive sheep are probably latently infected for life and harbor tissue cysts in various tissues. Therefore, the consumption of un- or undercooked meat from seropositive animals is a high risk for humans [2]. In the present study a type II strain was used for experimental infection of sheep since type II strains are most commonly found in animals in Europe and are most commonly identified as a cause of human toxoplasmosis world-wide [40]. A live intramuscular vaccine (Toxovax®, MSD Animal Health) that is on the market can prevent congenital toxoplasmosis in sheep. However, no current vaccine can prevent infection, suggesting that new vaccination strategies are needed. Since *T. gondii* infection of sheep mostly occurs via the oral route, inducing mucosal protection against the infection might be important. Therefore insights are needed in the acute events in the small intestine following oral infection [8,41]. Whereas several natural and experimental *T. gondii* infections have been performed in sheep, none focused in the same detail on the initial phase of the infection at the intestinal mucosa and lymphoid tissues and its local cytokine response as our study did [2,7,21,42-45].

In our study, sheep were orally infected with 3000 tissue cysts. Because infectious oocysts are more difficult to obtain in sufficient quantities, lead to environmental contamination and are a serious risk for laboratory personnel, tissue cysts where used even though oral infection in sheep mostly occurs via ingestion of oocysts during grazing [46]. Nevertheless infection with tissue cysts can occur, although rare, when sheep eat the placenta or abortion products from ewes that aborted as a result of toxoplasmosis [47-49]. As mentioned above, only a few studies analyzed the immune response in sheep, three of them after oral inoculation with oocysts [42,45,50] and four following an intraperitoneal injection with tachyzoites of the RH strain or S48 strain [17,51-53]. The latter is a non-natural infection. One other study analyzed the immune after a subcutaneous infection with tissue cysts, a non-natural infection route [54].

*T. gondii* tachyzoites preferentially entered the cranial small intestine around 4 dpi. Most likely, infection occurs at the same site when oocysts are given orally. Indeed, Dubey and Sharma [55] found that excystation of oocysts and the release of sporozoites takes place in the small intestine.

Since parasites were detected simultaneously in epithelial cells, lamina propria, MLN and blood in one sheep at 4 dpi, our findings suggest that some parasites most likely directly pass the epithelial barrier to reach MLN and blood. This early detection of parasite DNA in blood could be a consequence of the use of tissue cysts for oral infection, which might pass the epithelial layer more early than when infection occurs with oocysts, since Dubey and Sharma [55] detected parasitaemia between 6 and 11 days after orally inoculating sheep with oocysts [55]. Four days after infection, sheep were still IgM and IgG seronegative in IFA and IgG negative in TLA ELISA. However, stimulation of mesenteric lymphocytes and splenocytes from animals infected 4 days earlier induced a significantly higher IFN-gamma response than when tested later. The response at day 4 is too early to be the result of antigen-specific CD4+ and/or CD8+ T lymphocytes. A T-cell independent IFN-gamma production by NK-cells [56] and CD4+ T cells [57] has been described in mice and is dependent on the production of IL-12 and tumor necrosis factor-alfa (TNF-alfa) by macrophages [58]. In the present study, a generalized but low IL-12 production was measured in the MLN cultures at 4 dpi. During this early infection stage, the parasite triggers several components of the innate immune system. Macrophages, NK cells, dendritic cells as well as neutrophils release cytokines such as IL-12 and IFN-gamma as a result of the infection. This early produced IFN-gamma is most likely crucial for inducing resistance against the parasite [59,60]. Previous mouse studies have demonstrated that IL-12 production is critical to induce IFN-gamma production and protection against *T. gondii*. Indeed, in IL-12p35/p40 knockout mice, IFN-gamma levels were severely decreased and *Toxoplasma gondii* tachyzoites underwent uncontrolled replication, which most likely caused early death of the IL-12 deficient mice [61]. While triggering the innate immune system, parasite antigens are also taken up and presented by antigen-presenting cells, leading to a parasite-specific T-cell immunity [62].

At 8 dpi, parasite DNA was highly present in the tissues of the cranial small intestine and MLN and this remained so until 14 dpi, suggesting replication of the parasite in the small intestinal mucosa, mucosa-associated lymphoid tissues and associated draining lymph nodes. This is consistent with the observations of Dubey and Sharma [55] who found tachyzoites in the mesenteric lymph nodes of sheep from 7 dpi onwards. Also in mice an increase in parasite burden occurs in PP, MLN, IE and LP between 4 and 9 dpi [63]. Specific IgM and IgG antibodies appeared almost simultaneously 9 dpi,. Specific antibodies in the presence of complement can lyse and therefore clear extracellular tachyzoites [59]. Parallel with the appearance of antibodies, a significant decrease in the IFN-gamma response and a decrease in the IL-12 response were observed. Different antigens induced IL-12 in different gut tissues of different sheep. This was not the case for IFN-gamma. GRA7 quite consistently induced IFN-gamma in MLN lymphocyte cultures of the four animals tested at 8 and 10 dpi, whereas TLA clearly induced IFN-gamma in the PP lymphocyte culture of two of the four euthanized animals. This suggests that different antigens were involved in the induction of the IFN-gamma responses in PP versus MLN. Homing of the activated T cells from both lymphoid tissues to other sites could explain why both antigen preparations induced responses in PBMC and popliteal LN cell cultures. Such homing at 8 dpi is consistent with the increase of IFN-gamma producing CD4+ lymphocytes in efferent lymph of surgically cannulated experimentally infected lymph nodes at 11 dpi [52]. Our results suggest that a T cell independent response becomes replaced by an antigen-specific T lymphocyte response around 8 dpi. That several antigens induced IFN-gamma and IL-12 responses in popliteal lymph nodes and spleen cell cultures, distant systemic lymphoid tissues, supports for systemic migration of the parasite.

Our observation that the parasite DNA disappears from the small intestine, popliteal LN and PBMC and spleen 3 weeks after infection, indicates that the parasite load drops below the detection limit of the PCR. Indeed, a negative result in PCR does not imply that these tissues are completely free of parasite DNA. However, that most of the tested tissues became PCR negative could be an indication that the parasite is either suppressed in these tissues, cleared from these tissues or otherwise is leaving these tissues between 2 and 3 weeks post infection. This coincided with the significant increase of IgG antibodies between 9 and 21 dpi.

In previous studies, the immune response was studied following oral inoculation with oocysts or following intraperitoneal injection with tachyzoites of the RH strain [64,65]. Whereas antibodies were detectable 14 dpi following oral inoculation with 5000 or 50.000 oocysts [64], they were only detected 1 month post inoculation following intraperitoneal injection with tachyzoites [65]. In our study the oral inoculation with tissue cysts resulted in a more rapid antibody response. However, this response was similar to the one observed by McColgan et al. [54], which saw IgG appear nine days after experimental infection with tissue cysts [54]. This again supports the hypothesis that the parasite passes the intestinal epithelial barrier more quickly after an oral infection with tissue cysts, although it can not be excluded that the earlier response was a result of strain differences [8,14,55].

While the antibody response increased, and the parasite seems to disappear from systemic lymphoid tissues, also the IL-12 response in these tissues decreased or became absent. This coincided with a decreased or negative antigen-specific IFN-gamma response in PP, PBMC and popliteal LN cell cultures. Interestingly, these observations are in accordance with findings of Innes et al. [17,52] who infected sheep subcutaneously with tachyzoites. They could not detect IFN-gamma anymore in the lymph flow from lymph nodes draining the subcutaneous region from 11 days after infection onwards. At that time they noticed a switch from predominantly CD4^+^ proliferating lymphocytes to CD8^+^ proliferating lymphocytes. In our study, antigen-specific IFN-gamma responses were still present in MLN and spleen cell cultures 21 dpi. Since MLN not only drain the gut but also systemic sites such as the peritoneal cavity and since the spleen is the secondary lymphoid organ draining the blood both responses could play a role in controlling a systemic infection occurring at this stage [52].

## Conclusions

Our results indicate that parasites enter the cranial small intestine the first days after infection. Three weeks later *T. gondii* DNA could not be recovered anymore from the intestine. This coincided with the increase of IgG1 and IgG2 antibodies and a decrease of the antigen-specific IFN-gamma response in PP, PBMC and popliteal LN. Furthermore, the experiments support a collaborate role of humoral and cellular immunity in acute *T. gondii* infections in sheep.
